# Barriers and Facilitators of Exercise Participation Among Community-Dwelling Older Adults with Chronic Conditions: A Qualitative Study Using the COM-B Model and Theoretical Domains Framework

**DOI:** 10.3390/healthcare14121803

**Published:** 2026-06-22

**Authors:** Xiaoxiao Huang, Guochun Liu, Xiaoqian Xu, Xiaojing Li, Xiaofeng Yan, Wen Li, Huilin Shi, Xing Ming, Yuqing Xia, Shiqi Lu, Haolin Wei, Zhannuo Su, Shuqi Xin, Haobo Li

**Affiliations:** 1 College of Exercise Medicine, Chongqing Medical University, Chongqing 400016, China; 100674@cqmu.edu.cn; 2 School of Public Health, Chongqing Medical University, Chongqing 400016, China; 3 The Fifth Clinical College, Chongqing Medical University, Chongqing 400016, China; 4 School of Nursing, Chongqing Medical University, Chongqing 400016, China; 5 University of Leicester Joint Institute, Chongqing Medical University, Chongqing 400016, China; 6 School of Sport and Health, Shenyang Sport University, Shenyang 110102, China; 7 The Second Clinical College, Chongqing Medical University, Chongqing 400016, China; 8 School of Pediatrics, Chongqing Medical University, Chongqing 400016, China; 9 The First Clinical College, Chongqing Medical University, Chongqing 400016, China

**Keywords:** physical activity, multimorbidity, health behavior, behavior change, self-regulation, social support, primary healthcare, healthy aging

## Abstract

Background: In the context of population aging and the growing burden of chronic conditions, promoting exercise participation has become an important strategy for supporting healthy aging. However, older adults with chronic conditions often face multiple constraints related to symptom burden, risk perception, and everyday life. A theory-informed understanding of the determinants of exercise participation in this population is therefore needed. Methods: This study adopted a theory-informed qualitative descriptive design and conducted face-to-face semi-structured interviews with 30 community-dwelling older adults with chronic conditions. Purposive sampling was used to ensure variation in age, sex, chronic condition type, and exercise participation. Data were analyzed using the framework method guided by the Theoretical Domains Framework (TDF), and the resulting themes were subsequently mapped onto the Capability, Opportunity, Motivation–Behavior (COM-B) model. Results: Participants were aged 60–86 years, and most were women, had low educational attainment, came from rural backgrounds, and lived with multimorbidity. Participants described exercise participation as a day-to-day process of negotiating symptoms, risk, functional boundaries, and everyday responsibilities rather than as a simple matter of willingness. Although most participants recognized the value of exercise, many lacked disease-specific knowledge about suitable exercise types, safe intensity, progression, and warning signs. Symptom burden and functional limitations constrained exercise, but many participants used symptom-based self-regulation strategies, such as resting, slowing down, or modifying activity when discomfort occurred. Family members, peers, health professionals, and community resources could either facilitate exercise or restrict it, depending on their accessibility, continuity, specificity, and practical relevance. Continued participation was closely linked to perceived benefits, controllable risk, self-efficacy, positive emotional experience, and immediate bodily feedback. Conclusions: Exercise promotion for older adults with chronic conditions should move beyond general advice and provide disease-adapted exercise education, symptom-based self-regulation strategies, family and peer support, professional guidance, age-friendly community resources, and feedback mechanisms that support long-term maintenance.

## 1. Introduction

The world is undergoing rapid population aging. In 2020, the global population aged 60 and above exceeded one billion, and it is projected to increase to approximately 1.4 billion by 2030 and reach about 2.1 billion by 2050. During the same period, the population aged 80 and above is expected to increase to approximately 426 million by 2050 [1]. Population aging will enter an irreversible trend in the next decade [2]. Meanwhile, multimorbidity has become increasingly prevalent among older adults and poses a growing challenge for health systems. The global prevalence of multimorbidity among people aged ≥60 years is approximately 51.0% [3]. This structural change, coupled with the high burden of chronic diseases, has driven the healthcare system to shift from a “treatment-centered” service model to one that is “centered on long-term management and functional maintenance” [4,5].

There is a significant age gradient in physical inactivity worldwide. In 2022, the global prevalence of physical inactivity among people aged 60 and above was approximately 44.0%, significantly higher than the 31% rate among adults [6]. The risk of non-communicable diseases (NCDs) and functional decline attributed to insufficient physical activity have increased sharply [7]. The health and economic burden and scale of insufficient physical activity are constantly being quantified. In 2019, low physical activity was attributed to approximately 830,000 deaths and 15.75 million DALYs worldwide, and this burden increased with age [8,9]. If the global level of insufficient physical activity does not improve, the public health care system will bear an additional cost of approximately 300 billion US dollars for related diseases between 2020 and 2030 [10]. If the level of insufficient physical activity remains unchanged, approximately 499.2 million new preventable cases of major NCDs could be added globally by 2030, generating about INT$520 billion in direct health care costs [11]. In terms of productivity loss, macroeconomic model projections show that even with a slight increase in physical activity levels at the population level, global GDP could be approximately 138 to 338 billion US dollars higher by 2025 compared to the current scenario, and about 314 to 760 billion US dollars higher by 2050. Among them, the contribution of “presenteeism” was reduced by approximately 70% [11,12]. These pieces of evidence support that physical activity promotion can save medical resources and also affect macroeconomic performance through effective labor supply and on-the-job efficiency.

In China, approximately 71% of the adults aged 60 years and above do not engage in moderate to high-intensity physical activities, and the proportion of those with a relatively low overall physical activity level can reach 75% [13]. In China, the coexistence of multiple diseases increases rapidly with age. A systematic review shows that the prevalence rates among people aged 60–69, 70–79 and ≥80 were 32.4%, 38.5% and 40.2% respectively, suggesting that the management of chronic diseases and the promotion of physical activity must be advanced simultaneously in old age [14]. This highlights that older adults with chronic conditions are precisely the high-risk group that most needs to improve their functions, control the course of the disease and reduce complications through exercise. Physical exercise is regarded as an important “prescription” for secondary prevention of chronic diseases, functional maintenance and fall risk management [15]. Systematic evidence supports the inclusion of exercise as a standard component in the management of 26 types of NCDs, including cardiovascular, metabolic, respiratory, musculoskeletal, neurological and oncology [16]. However, health promotion strategies that merely advocate but do not implement are difficult to reverse behavioural outcomes and ultimately fail to bring about health gains [6]. The participation rate and compliance of older adults with chronic conditions in physical exercise are still insufficient. The determining factor is not a single issue of “willingness”, but the comprehensive result of multiple constraints such as capability, opportunity and motivation [17].

The participation obstacles in physical exercise among older adults with chronic conditions present a multi-layered coupling. Personal-level obstacles include fear of injury or worsening of illness, chronic pain and physical discomfort, low motivation, limited interest, and perceived lack of time [18]. Social and environmental factors also influence exercise participation among older adults. Family care responsibilities, insufficient social support, as well as conditions such as accessibility, cost, season/weather, and venue safety jointly determine “whether there are feasible opportunities” [19]. It is worth noting that many elements have situational bidirectionality. Peer/family support, community resources and organizational forms may either become obstacles or transform into promoting factors under different conditions, suggesting that multifactorial interventions are needed rather than approaches limited to health education alone [20]. A growing body of qualitative research and systematic reviews has clarified barriers and facilitators of physical activity participation among older adults [18,20,21]. However, “listing factors” alone is still not sufficient to answer why older adults with chronic conditions remain inactive, why they are active, and how they can shift from occasional participation to long-term persistence. It is therefore necessary to systematically classify these determinants using behavioural science frameworks and translate them into actionable intervention targets [21]. A more translational approach is to use the Behavior Change Wheel as a methodological basis and COM-B as an integrative framework, with the TDF providing a more granular structure for coding behavioural determinants [17,22]. However, theory-driven research on physical activity among older adults with chronic conditions in China remains limited, and the behavioural mechanisms underlying exercise participation remain insufficiently explored.

Based on this, this study adopts a qualitative research design guided by theory, with COM-B and TDF as the main analytical lines. This study used semi-structured interviews to explore barriers and facilitators of exercise participation among community-dwelling older adults with chronic conditions, clarify behavioural mechanisms within individual, family, and community contexts, and identify priority targets for future intervention development.

## 2. Methods

### 2.1. Research Design

This study adopted a descriptive qualitative research design guided by theory [23], to explore barriers and facilitators of exercise participation among older adults with chronic conditions in the community through semi-structured one-on-one interviews. Qualitative description was selected to provide a close-to-data account of participants’ experiences, perceptions, and contextual influences related to exercise participation.

The Capability, Opportunity, Motivation–behavior model (COM-B) was used as the overarching behavioural framework, and the Theoretical Domains Framework (TDF) was used to identify and categorise more specific determinants of exercise participation [17,24]. COM-B and TDF were applied as a priori sensitising and organising frameworks rather than as inductively generated themes. They informed the development of the interview guide, the construction of the initial coding framework, and the interpretation of findings in relation to potential intervention targets [25]. Data were analysed using the framework method [26]. Transcripts were first coded deductively according to relevant TDF domains, while data that did not fit the initial framework were retained through inductive coding. Codes were then compared, refined, and grouped into themes, which were subsequently mapped onto the COM-B components to support higher-order interpretation. Reporting followed the Consolidated Criteria for Reporting Qualitative Research (COREQ) [27].

### 2.2. Participants

Participants were community-dwelling older adults with chronic conditions. Inclusion criteria were: (1) age 60 years or older; (2) diagnosis of at least one chronic non-communicable disease by a medical institution, with a stable condition in the previous 3 months and the capability to perform activities of daily living independently; (3) no severe cognitive impairment or acute mental disorder, and the capability to understand the interview questions and complete the interview; and (4) voluntary participation with written informed consent. Exclusion criteria were: (1) acute exacerbation or recent severe complications; (2) explicit medical advice against exercise or unsuitability for interview participation; and (3) communication barriers that prevented data collection. Purposive sampling was used to capture variation in age, sex, chronic condition type, multimorbidity status, exercise participation, functional status, and living arrangement. Potential participants were identified with the assistance of community administrators and were screened for eligibility by trained researchers. Eligible older adults were then approached face-to-face at the community service center for older adults, informed about the study purpose and procedures, and invited to participate. Recruitment and preliminary analysis proceeded concurrently. After 26 interviews, no new substantive codes, TDF domains, or major themes relevant to exercise participation were identified. Four additional interviews were conducted to assess the stability of the coding framework across participants with different chronic conditions and exercise experiences. Informational saturation was considered achieved when these interviews generated no new analytically important determinants. Because some potential participants were initially contacted informally by community administrators before researcher screening, the number who declined at this preliminary stage was not systematically recorded. No participant withdrew after providing informed consent.

### 2.3. Interview Outline Design

The semi-structured interview guide was developed from three sources: previous qualitative studies and reviews on physical activity or exercise among older adults and people with chronic conditions, the COM-B model, and the TDF. COM-B informed the broad domains of questioning, including capability, opportunity, and motivation, while TDF informed more specific prompts related to knowledge, physical skills, behavioural regulation, environmental context and resources, social influences, beliefs about consequences, beliefs about capabilities, emotion, and reinforcement. A social ecological perspective was also considered to ensure that questions captured individual, interpersonal, professional, community, and environmental influences. The draft guide was reviewed by two experts in public health and geriatrics, and minor wording revisions were made to improve clarity and acceptability for older adults with low educational attainment [17,25]. The interview guide covered: (1) capability-related factors, including health status, physical or cognitive difficulties, knowledge of exercise benefits and risks, and perceived ability to perform appropriate exercise; (2) opportunity-related factors, including family and peer support, professional guidance, community venues, equipment, cost, accessibility, and weather-related constraints; and (3) motivation-related factors, including perceived benefits and risks, willingness to participate, confidence, emotional experience, and strategies for maintaining exercise [21,28,29,30]. The interview guide is provided in the Supplementary Material.

### 2.4. Data Collection

Data were collected between 10 January and 28 February 2026, at a community service center for older adults in Chongqing, China. Interviews were guided by the semi-structured interview guide described above and were conducted face-to-face in a quiet room at the center. Before each interview, participants received information about the study purpose, the researcher’s role, confidentiality, voluntary participation, and their right to withdraw. Written informed consent was obtained and orally reconfirmed before audio-recording [27].

Interviews were conducted by two female postgraduate researchers: X.X., with a background in clinical medicine, and X.L., with a background in nursing. Both interviewers received standardised training in qualitative interviewing, research ethics, confidentiality, use of the interview guide, and communication with older adults. Neither interviewer had a prior clinical, family, social, or research relationship with the participants. To reduce potential interviewer influence, open-ended prompts were used, corrective or educational responses were avoided during interviews, and reflexive field notes were recorded after each interview.

Each interview was conducted by one interviewer with one recorder present. Interviews lasted 30–60 min, were audio-recorded, transcribed verbatim in Chinese, anonymised, and checked against the recordings. Field notes documented non-verbal cues, contextual observations, and interviewer reflections. For participants with visual or literacy difficulties, questions were read aloud and comprehension was checked before responses were recorded. Sociodemographic and health-related information was collected after the interview.

Full transcripts were not routinely returned because several participants had low literacy or preferred oral communication. Instead, summary checking was conducted during or immediately after interviews, allowing participants to clarify or correct key points. Quotations selected for publication were translated into English by bilingual team members and reviewed to preserve participants’ core meanings, contextual information, and intended views. Minor grammatical edits were made solely for readability and did not alter the substantive meaning.

### 2.5. Data Analysis

Data were analysed using the framework method. We first deductively coded interview data to relevant TDF domains. Two researchers (X.L. and X.X.) independently coded an initial subset of transcripts to refine the coding framework and ensure consistent application of TDF domains. Coding disagreements were resolved through discussion and review of the original transcripts. After agreement was reached, subsequent transcripts were coded using the refined framework. We remained open to inductive coding when data did not fit the initial TDF framework, to ensure that context-specific determinants of exercise participation were not missed. Codes within each TDF domain were compared and grouped into themes. The themes were then mapped onto the COM-B components to support higher-order interpretation of exercise participation behavior. NVivo 12 (QSR International Pty Ltd., Doncaster, Australia) was used for data management, coding and framework matrix organisation [31]. Regular analytic meetings were held to compare interpretations, discuss discrepant cases, and refine theme development and COM-B mapping. Disagreements were discussed until consensus was reached, and unresolved disagreements were adjudicated by G.L. Analytic memos documented preliminary interpretations, reflexive notes, and coding decisions, while the audit trail recorded codebook revisions, theme development, COM-B mapping decisions, and exemplar quotations. Participants did not review the final thematic structure; credibility was supported through summary checking during data collection, multiple analysts, analytic memos, and transparent documentation of analytic decisions. Coverage was reported only to indicate the breadth of thematic reach across participants and should not be interpreted as prevalence, importance ranking, or effect size.

### 2.6. Ethics

This research protocol was approved by the Medical Research Ethics Committee of Chongqing Medical University (Approval Number: 2026-085). All participants signed the informed consent form. The research strictly adheres to medical ethics guidelines and the requirements of the Declaration of Helsinki, protecting the privacy and confidentiality of the participants’ information.

## 3. Results

### 3.1. Overview of the Study Sample

A total of 30 participants were included in this study, aged between 60 and 86 years old. Among them, 21 were female (70.0%) and 9 were male (30.0%). Overall, the participants were characterised by low educational attainment, rural backgrounds, and a high proportion of multimorbidity. Seventeen (56.7%) participants had a primary school education or below, 11 (36.7%) had a junior high school education, and 2 (6.7%) had a senior high school education or above. Seventeen participants (56.7%) were from rural areas, and 13 (43.3%) were from cities or county towns. In terms of living patterns, 12 (40.0%) lived with their children or grandchildren, 9 (30.0%) lived alone, 7 (23.3%) lived with their spouses, and 2 (6.7%) lived with their spouses and children or grandchildren. In terms of health status, 22 participants (73.3%) were living with multimorbidity, defined in this study as the coexistence of two or more chronic conditions, and 23 (76.7%) reported long-term or continuous medication use. The most commonly reported chronic conditions included hypertension, diabetes, musculoskeletal conditions (including lumbar disc disease, sciatica, and chronic joint pain), chronic respiratory conditions (e.g., asthma), cerebrovascular disease/stroke, gastrointestinal conditions, and sensory impairment (see Table 1).

### 3.2. Overview of Themes and Framework Mapping

TDF-guided framework analysis identified interrelated determinants of exercise participation, which were subsequently mapped onto COM-B components to support higher-order interpretation. COM-B was used as an analytic mapping framework rather than as an emergent finding. Overall, exercise participation was best understood as a dynamic process in which older adults negotiated symptoms, perceived risk, functional boundaries, social support, environmental conditions, and expected or experienced outcomes.

Within the capability component, participants described experiential but incomplete exercise knowledge, unclear disease-specific risk boundaries, symptom-based self-regulation, and functional limitations related to chronic conditions. Within opportunity, exercise participation depended on whether family members, peers, clinicians, and community resources could be translated into practical and sustained opportunities for action. Within motivation, decisions to continue, modify, or avoid exercise were shaped by perceived benefits and risks, self-efficacy, emotional responses, and reinforcing bodily or daily-life feedback. Coverage is reported only to indicate the breadth of thematic reach across participants and should not be interpreted as prevalence, ranking, or effect size (Table 2).

### 3.3. Capability

#### 3.3.1. Psychological Capability: Knowledge and Understanding (TDF: Knowledge)

Within psychological capability, participants were generally aware that exercise could be beneficial, but their knowledge was mainly experiential, lay, and non-specific. They often judged the appropriateness of exercise through bodily sensations, such as comfort, sweating, fatigue, pain, or breathlessness, rather than through disease-adapted guidance. Some participants associated exercise with better sleep, breathing, mobility, or lower-limb function; however, few described clear knowledge of suitable exercise types, safe intensity, progression, or warning signs in relation to their chronic conditions.

*I have mastered the correct way of exercising. One needs to arrange the amount of exercise based on their own situation, but I’m not sure if it’s correct or not. It’s more about treating exercise as a personal hobby. (p14, female, multimorbidity)*

*I think as long as you walk and move, it’s considered exercise. (p28, male, multimorbidity)*

#### 3.3.2. Psychological Capability: Behavioural Regulation and Action Routines (TDF: Behavioural Regulation; Memory, Attention and Decision Processes)

##### Behavioural Regulation

Although some participants did not have a formal exercise plan, many had developed practical self-regulation strategies in daily life. They adjusted their exercise rhythm according to bodily feedback, such as pain, breathlessness, fatigue, or discomfort, and maintained activity by walking at fixed times, resting when necessary, or carrying medication. This suggests that capability was not only related to exercise knowledge, but also to whether participants could adapt activity safely under fluctuating symptoms.

*When I’m not feeling well, I won’t exercise. But when I have no symptoms, I’ll do more exercise (p3, female, multimorbidity)*

*Sometimes when I feel a bit painful during exercise, I’ll rest. (p7, female, multimorbidity)*

##### Memory, Attention and Decision Processes

The TDF domain of memory, attention, and decision processes was reflected in whether exercise was embedded in daily routines. A small number of participants had established regular patterns, such as walking at fixed times or attending square dancing, which reduced the need for repeated decision-making. In contrast, when exercise was not linked to a stable routine, it was more likely to be interrupted by physical discomfort or daily affairs.

*Usually, I start taking a walk at around 8 o ‘clock and walk until after 10 o ‘clock. In the afternoon, I go for another walk. (p26, female, multimorbidity)*

*I don’t have a fixed exercise time. I exercise whenever I want. (p16, female, asthma)*

#### 3.3.3. Physical Capability: Symptom Burden and Functional Limitations (TDF: Physical Skills)

Chronic symptom burden and physical function limitations were recurrent barriers within the capability dimension, mainly manifested as pain, shortness of breath, weakness, vision decline and lower limb function limitations. These problems did not necessarily prevent exercise completely, but they reduced the duration, intensity, and range of feasible exercise options, making exercise more likely to be low-intensity, intermittent and highly individualized. Some participants could only maintain their activities by walking slowly, resting, and then walking again, while others avoided going out at night and new forms of exercise due to vision problems, weak knees and legs or fear of falling.

*I can’t stand for too long because my knees are not in good condition. (p10, female, multimorbidity)*

*I don’t dance anymore now for fear of falling. (p22, female, multimorbidity)*

### 3.4. Opportunity

#### 3.4.1. Physical Opportunity: Environmental Context and Resource Accessibility (TDF: Environmental Context and Resources)

Physical opportunity was reflected in whether the community environment was convenient, age-friendly, and sustainably usable. Some participants considered the community environment generally convenient and accessible. The nearby parks, trails or basic equipment can meet the needs of daily walking, jogging and simple exercises. For these participants, accessible activity spaces lowered the threshold for going outdoors and helped integrate exercise into daily life.

*The community is quite suitable for taking a walk and the facilities are all very good. (p14, female, multimorbidity) The community can be equipped with age-friendly exercise facilities. The current facilities are quite suitable for young people. (p7, female, multimorbidity)*

However, participants more often emphasised the gap between the mere availability of resources and their suitability for older adults with chronic conditions. Weather changes, road conditions, equipment damage, lack of indoor alternative venues and excessive distance can all directly reduce the opportunities for exercise. For older adults with chronic conditions, these environmental barriers were not merely minor inconveniences but can be combined with their pain, shortness of breath, urgency, poor balance and concerns about falls, thereby forcing them to shorten, reroute or interrupt their exercise.

*My condition is greatly affected by seasonal weather, and there are no indoor exercise venues. (p27, male, stroke) There is no exercise equipment in the community… I would be willing to participate in seated exercises. (p28, male, multimorbidity)*

#### 3.4.2. Social Opportunity: Social Support, Companionship and Professional Guidance (TDF: Social Influences)

Within social opportunity, family members, peers, and professional support jointly shaped exercise participation among older adults with chronic conditions. Some participants received positive support from family members, peers or doctors. This support was manifested in verbal encouragement, joint activities, risk warnings and moderate exercise advice, which helped them start or maintain exercise. For participants who were older, lived alone, or had multimorbidity, companionship provided not only emotional support but also a sense of safety.

*My daughter and granddaughter are very supportive of my exercise and even exercise with me. (p5, female, hypothyroidism)*

*I haven’t received a doctor’s advice on exercise. (p23, female, multimorbidity)*

However, social support did not always translate into actionable opportunities for exercise. Some family members, although supportive in attitude, lack the time to accompany them. Some other family members tend to dissuade them out of fear of falls, recurrence of old injuries or aggravation of the condition. Meanwhile, the exercise guidance provided by medical staff and the community is often rather limited. Participants repeatedly described a lack of organised guidance, timely notification, and specific professional advice, suggesting that the lack of social opportunities is not only manifested in weak support from relatives, but also in insufficient professional and organizational support. Participants rarely distinguished exercise professionals from other healthcare or community personnel, suggesting that available professional guidance was perceived mainly as general advice rather than disease-adapted exercise support.

*I haven’t heard of it. If there were a notice, I would still be willing to participate… I hoped they would come to help me, but they didn’t. (p4, female, multimorbidity)*

*My family said that I had a history of falls, and they suggested that I should stay at home more… But I still like going out for a walk. (p11, female, multimorbidity)*

Within this broader context of social influence, family responsibilities and everyday role expectations further shaped the priority given to exercise. Some participants were willing to exercise but had to prioritise childcare, housework, and other family responsibilities. For these participants, exercise depended partly on whether everyday obligations left sufficient time, space, and practical room for action.

*Before going out to exercise, I’ll make sure my children are well arranged. (p14, female, multimorbidity)*

*I help my daughter take care of my granddaughter, so I don’t have much time to exercise. (p12, female, multimorbidity)*

### 3.5. Motivation

#### 3.5.1. Reflective Motivation: Beliefs About Consequences, Beliefs About Capabilities, and Intentions/Goals (TDF: Beliefs About Consequences; Beliefs About Capabilities; Intentions/Goals)

##### Beliefs About Consequences

Within reflective motivation, beliefs about consequences shaped whether participants viewed exercise as worth continuing. These beliefs were bidirectional. Some participants associated exercise with better sleep, mood, lower-limb function, or general health, which strengthened positive expectations. Others associated exercise with falls, injury, discomfort, or symptom aggravation, leading to caution or avoidance.

*I do worry about bumping into something or feeling uncomfortable during exercise. (p17, male, chronic gastritis) After exercising, I feel better, my time feels more fulfilling, and I can also lose weight. (p15, female, multimorbidity)*.

##### Beliefs About Capabilities

Participants differed in their confidence to exercise safely under current health conditions. Some reported reduced confidence because of advanced age, chronic symptoms, or previous injury, and therefore limited continuous exercise or avoided new activities. Others remained confident in maintaining familiar activities by adjusting pace, duration, or intensity. Thus, capability beliefs were less about whether participants could move at all, and more about whether they believed they could exercise safely and sustainably within their current functional boundaries.

*If my physical condition permits, I will still go and dance in the square. (p4, female, multimorbidity)*

*I usually dance, but I haven’t tried traditional forms of exercise and have no interest in them. I’m getting older and do not feel motivated to try new activities. (p24, female, multimorbidity)*

##### Intentions/Goals

At the level of intentions and goals, most participants did not describe explicit exercise goals or structured plans. Their intentions were usually conditional and practical: they were willing to continue familiar activities or attend organised sessions if these were perceived as useful, affordable, and compatible with daily responsibilities. Thus, intention was present, but it was often expressed as conditional willingness rather than proactive goal setting or planned progression.

*I haven’t heard of any training with exercise guidance. If there is a notice, I would still be willing to participate. (p4, female, multimorbidity)*

*I haven’t considered having a doctor or exercise professionals work with me to make an exercise plan. (p17, male, chronic gastritis)*

#### 3.5.2. Automatic Motivation (TDF: Emotion; Reinforcement)

##### Emotion

Within automatic motivation, emotional experience influenced whether participants were willing to continue exercising. For some participants, exercise was not only a way to maintain health but also a source of enjoyment, emotional relief, and daily fulfilment. Positive affect increased the subjective appeal of exercise, whereas fear, worry, or limited positive experience could lead to avoidance.

*Exercise improves mood, makes people happier and makes time more fulfilling. (p14, female, multimorbidity) Exercise can make life feel more fulfilling and one’s mood will also be better. (p13, male, multimorbidity)*

##### Reinforcement and Habit Formation

Within reinforcement, immediate bodily feedback after exercise and simple self-monitoring together appeared to support continued participation. Some participants were able to experience direct physical comfort or improvement in their condition after the activity, which reinforced the belief that exercise was worth continuing. Others used WeChat step counts or daily step records as visible feedback. These findings suggest that reinforcement came not only from general health knowledge, but also from perceived bodily benefits and trackable behavioural feedback.

*Record my steps every day (p5, female, hypothyroidism)*

*Doing some aerobic exercises makes my body feel very relaxed. (p2, female, multimorbidity)*

## 4. Discussion

This study shows that exercise participation among community-dwelling older adults with chronic conditions is not simply a matter of willingness, but a process of risk management and supported self-regulation under chronic-condition constraints. The central knowledge gap was not a lack of awareness that exercise is beneficial, but insufficient operational knowledge about how to adapt exercise to symptoms, risk, and functional boundaries. Social support was also conditional: it could enable exercise through companionship and safety support, but could restrict participation through protective dissuasion or non-specific advice. These findings position exercise promotion as a problem of disease-adapted capability building, opportunity creation, and motivational reinforcement, rather than general health education alone [21,30]. For example, a participant who could walk only for several minutes before lumbar or knee discomfort would require short-bout walking, planned rest breaks, and clear symptom-based stopping rules rather than generic advice to “walk more”; similarly, a participant who avoided exercise because of fear of falling would require safer timing, accompaniment, or indoor alternatives rather than motivational encouragement alone.

First, at the capability level, this study reveals a critical gap: participants do not simply lack exercise awareness. Instead, they lack disease-specific exercise knowledge. Specifically, they struggle to identify suitable activities, determine safe intensity levels, manage discomfort, and adjust routines during symptom fluctuations. Existing reviews also suggest that older adults often have a basic understanding of the value of physical activity, but their understanding of specific implementation methods, risk boundaries, and individualized adjustments is often insufficient. This state of “knowing it is beneficial but not implementing it” will weaken the initiation and persistence of behavior [19,21,30]. One of the reasons might be that the overall educational attainment and health literacy among older adults in China are relatively limited. Among 10,749 older adults aged 60 and above, 54.3% of the participants have an educational level lower than primary school [32]. Although the health literacy of older adults in China has improved, it is generally low and shows urban-rural differences [33]. Therefore, although some older adults with chronic conditions recognize the benefits of exercise, they may not have the capability to translate general health knowledge into safe, concrete and sustainable exercise behaviours. This is consistent with the direction of the association between health literacy and health outcomes as observed in the national representative survey [34].

Meanwhile, a large number of participants developed dynamic adjustment strategies based on symptom feedback, such as reducing the intensity in a timely manner according to pain, shortness of breath, dizziness or palpitations, and resuming activities after resting. This indicates that symptom-driven behavioural regulation should not be simply regarded as “non-persistence” or “poor compliance”, but should be understood as an adaptive mechanism for risk management in the real life of older adults with chronic conditions [21,22,30,35,36]. Therefore, from an intervention-development perspective, this finding can be translated into specific, executable, and self-monitorable exercise rules, including a safe starting threshold, symptom warning signals, intensity-adjustment methods, alternative activities during symptom fluctuation, and a restart pathway after interruption [37,38]. Operationally, these rules could be formalized as a symptom-contingent activity-pacing protocol, linking symptom severity and stability to exercise continuation, dose modification, temporary cessation, and medical review.

Second, at the opportunity level, this study did not simply define family, peers, medical staff and community resources as “supported” or “unsupported”, but rather demonstrated that these factors have obvious bidirectionality. The same social support may either enhance a sense of security and the possibility of action in different situations, or it may become a source of behavioural resistance due to excessive worry, lack of companionship, insufficient information or organizational discontinuity [19]. Similarly, the role of the community environment does not merely depend on “whether there is a venue or not”, but more on “resource adaptability” such as the availability of equipment, the safety of the space, its accessibility, and whether there are alternative scenarios when the weather changes [20,30]. This result is consistent with the view in the research on the physical activity of older adults that “quality is more important than existence” in social support, because what truly promotes physical activity is often not abstract attitude approval, but rather reminders, companionship, feedback, joint participation and available specific assistance directly related to exercise, especially support from family members, which has a more realistic impact [39].

For older adults with chronic conditions, this two-way nature is even more prominent. They face not only the general decline in physical fitness associated with aging, but also specific concerns regarding falls, symptom recurrence, and the compounded risks of multimorbidity. Therefore, both the “protective dissuasion” from family members and the “generalized advice” from medical staff may unintentionally compress their exercise boundaries [18,36]. our findings show that insufficient social opportunities are not only reflected in limited family support, but also in the failure of professional and organizational support to be steadily transformed into sustainable exercise opportunities. This is consistent with the findings from the investigation and research that although medical staff generally hold a positive attitude towards promoting physical activity among older adults, there are deficiencies in terms of knowledge, conventional discussion paths, and specific implementation plans [39,40]. For older adults with chronic conditions, sustained exercise participation may require an integrated community–primary care support pathway that combines brief exercise counselling, family risk communication, referral to appropriate community resources, peer-supported activity, and context-sensitive alternatives for environmental or symptom-related constraints. Given the characteristics of living alone, disability, low educational attainment and coexistence of multiple diseases, future social intervention should pay more attention to the fact that physical activity intervention provided or triggered by health professionals in primary care can improve activity levels. This provides a practical basis for incorporating exercise support into the routine management of chronic diseases [40,41,42].

Third, at the motivation level, this study found that the participants did not have a binary question of “willing to exercise” and “unwilling to exercise”, but rather were closer to a continuous benefit-risk trade-off. When participants believe that exercise can improve sleep, mood, leg and foot function or overall condition, exercise is more likely to be incorporated into their daily lives. However, when they associate exercise with falls, injuries, aggravated symptoms or excessive fatigue, avoidance and self-restraint are more likely to occur [19,30]. Existing evidence indicates that older adults’ judgements about the value of physical activity are often not based on abstract health slogans, but rather on a realistic assessment of whether they can truly feel the benefits, whether they are worried about injury, and whether the activity is in line with their current physical condition [30,39]. Meanwhile, self-efficacy plays a pivotal role in this process. When individuals believe they can safely move around in their current health status, their behavioural intentions are more likely to translate into sustained participation. When a sense of age, past negative experiences or functional decline are internalized as the judgment of “I can’t do it anymore”, behavior is more likely to be restricted or discontinued [43,44]. Therefore, among older adults with chronic conditions, motivation is not an internal psychological state separated from the disease, but a cognitive-emotional comprehensive evaluation process embedded in symptom experience, past events and social feedback. This is also why simply emphasizing “enhanced awareness” is often insufficient to bring about stable behavioural changes. Furthermore, this study suggests that emotional experience and reinforcement feedback are of great significance for the long-term maintenance of exercise. Some participants experienced a sense of relaxation, pleasure and a “more fulfilling time” after exercising. Such immediate positive feedback constitutes an important source of reinforcement for continuous participation. On the contrary, if one does not feel significant improvement for a long time or is constantly dominated by fear and anxiety before and after exercising, it is more likely to develop an avoidance tendency. Existing studies have shown that physical activity acceptance and maintenance among older adults are often closely related to pleasure, satisfaction, perceived benefits and positive experiences after activity, rather than merely depending on long-term disease prevention information [45,46]. Motivational support should therefore target self-efficacy and maintenance through graded mastery, affective reinforcement, and low-burden self-monitoring, while framing symptom-adjusted participation as adaptive self-regulation rather than non-adherence.

Based on these findings, this study has clear implications for intervention development. According to COM-B and TDF, intervention should not remain at the level of general health education, but should target specific behavioural determinants. At the capability level, interventions should strengthen disease-adapted exercise education and symptom recognition training. At the opportunity level, they should improve age-friendly community environments and translate family and peer support into companionship, reminders, and safety support. At the motivation level, they should enhance perceived benefits, strengthen self-efficacy, reduce excessive risk perception, and provide reinforcing feedback [17,25]. Behavior change techniques such as education, goal setting, self-monitoring, prompts and cues, feedback, social support, and environmental restructuring may therefore be used to develop more targeted and evaluable community-based exercise promotion programmes.

Taken together, these findings identify actionable targets for intervention development. Capability-focused components should integrate disease-adapted education with symptom recognition and self-regulation to support safe exercise initiation, titration, temporary cessation, and resumption. Opportunity-focused components should coordinate family, peer, clinical, and community resources to convert social concern and environmental constraints into supported participation. Motivation-focused components should strengthen self-efficacy and maintenance through early mastery experiences, positive affect, and feedback-based reinforcement. These components could be embedded in community exercise programmes, chronic-disease follow-up, and primary healthcare, with evaluation centred on exercise initiation and maintenance, symptom-management confidence, perceived safety, and quality of life.

## 5. Limitations

This study has several limitations. Firstly, the sample was drawn from specific community contexts and was mainly composed of women, those with rural backgrounds, those with low educational attainment, and those with multimorbidity. The findings are therefore most transferable to similar community care contexts, and transferability to other regions, cultural settings, and service models should be assessed cautiously. Second, although purposive sampling was used to capture variation in age, sex, chronic condition type, exercise participation, functional status, and living arrangement, some subgroups may still have been under-represented. Thirdly, this study uses COM-B and TDF as the prior framework, which is helpful to improve the interpretive focus and intervention translatability, but it may also affect the breadth of open coding to a certain extent. Finally, coverage was reported only to indicate the breadth of thematic reach across participants and should not be interpreted as prevalence, importance ranking, or effect size. Future quantitative research is still needed to examine the relative salience of different determinants and their relationship with behavioural outcomes.

## 6. Conclusions

Exercise participation among community-dwelling older adults with chronic conditions is shaped by the continuous interaction of capability, opportunity, and motivation within the realities of symptoms, perceived risk, functional boundaries, family roles, and community resources. The findings suggest that exercise promotion for this population should move beyond general advice and provide disease-adapted knowledge, symptom-based self-regulation strategies, family and peer support, professional guidance, age-friendly community environments, and reinforcing feedback. By mapping qualitative findings onto COM-B and TDF, this study identifies actionable behavioral determinants that can inform the development of targeted, acceptable, and evaluable exercise promotion interventions in community care, chronic-disease management, and primary healthcare practice.

## Figures and Tables

**Table 1:** Sociodemographic and health-related characteristics of participants.

Characteristic	n (%)
Age group, years	
60–69	11 (36.7)
70–79	11 (36.7)
≥80	8 (26.7)
Gender	
Male	9 (30.0)
Female	21 (70.0)
Educational attainment	
Primary school and below	17 (56.7)
Junior high school/Incomplete junior high school	11 (36.7)
High school	2 (6.7)
Household registration	
Rural areas	17 (56.7)
City/County Town	13 (43.3)
Living arrangement	
Living alone	9 (30.0)
Living with spouse	7 (23.3)
Living with children/grandchildren	12 (40.0)
Living with spouse and children/grandchildren	2 (6.7)
Health-related characteristics	
Long-term medication	23 (76.7)
Participants with multimorbidity (≥2 chronic conditions)	22 (73.3)

**Table 2:** Themes identified through TDF-guided framework analysis and their mapping onto the COM-B model.

COM-B Component	TDF Domain	Theme	Functional Role	Coverage, n (%)	Typical Quotations
Capability—Psychological capability	Knowledge	Experiential exercise knowledge and unclear disease-specific risk boundaries	Bidirectional	26 (86.7)	If there are appropriate methods that are beneficial for my exercise, but I have not yet mastered them. (p30, male, multimorbidity) I exercise when I feel able to do so, and I stop when I feel unable to continue (p21, male, multimorbidity)
	Behavioural regulation	Symptom-based self-regulation and contingency planning	Bidirectional	28 (93.3)	If my heart beats too fast, I will rest on the spot. (p23, female, multimorbidity) Sometimes when I get tired from walking, I take a break and then continue walking. (p18, female, multimorbidity)
	Memory, attention and decision processes	Habitual routines and simple adjustment strategies	Facilitator	4 (13.3)	Every morning I get up and ride a bike, and in the evening I go to square dancing. (p20, male, multimorbidity)
Capability—Physical capability	Physical skills	Symptom burden and functional limitations	Barrier	18 (60.0)	I can only walk for five or six minutes at most before I feel swelling and pain in my lumbar spine and knees. (p3, female, multimorbidity)
Opportunity—Physical opportunity	Environmental context and resources	Environmental accessibility, safety, and age-friendliness	Bidirectional	10 (33.3)	Some exercise equipment is broken and can no longer be used. (p25, male, diabetes) The nearby environment is generally suitable for walking. (p12, female, multimorbidity)
Opportunity—Social opportunity	Social influences	Social support, companionship and professional guidance	Bidirectional	17 (56.7)	I think exercise is good, but my children are busy with work every day and cannot accompany me. (p6, female, chronic gastritis) My family reminds me not to overdo it and to adjust exercise according to my physical condition. I should not jump around. (p29, male, multimorbidity)
Motivation—Reflective motivation	Beliefs about consequences	Perceived benefits and anticipated harms of exercise	Bidirectional	4 (13.3)	I worry about bumping into something or feeling uncomfortable during exercise. (p17, male, chronic gastritis) After exercising, I feel better, my time feels more fulfilling, and I can also lose weight. (p15, female, multimorbidity).
	Beliefs about capabilities	Confidence in exercising safely under current conditions	Bidirectional	23 (76.7)	I do not want to exercise anymore. After all, I’m already 80 years old. (p7, female, multimorbidity) I am confident about walking. I dare to walk alone; I am not afraid. I just need to walk slowly. (p1, female, multimorbidity)
	Intentions/Goals	Conditional intention and limited action planning	Bidirectional	28 (93.3)	If there is a notice, I would still be willing to participate. (p4, female, multimorbidity) I have not considered asking a doctor or exercise professional to develop an exercise plan for me. (p17, male, chronic gastritis)
Motivation—Automatic motivation	Emotion	Negative emotions and low positive feedback	Barrier	11 (36.7)	I am afraid of falling at night and having no one to help me. (p21, male, multimorbidity)
	Emotion	Positive emotional experience	Facilitator	15 (50.0)	I feel very happy when square dancing. (p19, female, multimorbidity)
	Reinforcement	Reinforcing feedback and self-monitoring	Bidirectional	21 (70.0)	Doing aerobic exercise makes my body feel very relaxed. (p2, female, multimorbidity) I walk every day, but my mood does not change much and my sleep quality is still poor. (p28, male, multimorbidity).

Note: Coverage indicates the proportion of participants who mentioned each theme among the 30 participants and is reported only to show the breadth of thematic reach. It should not be interpreted as prevalence, importance ranking, or effect size. “Bidirectional” indicates that the same theme could function as either a barrier or a facilitator depending on the context. For bidirectional themes, quotations were selected to illustrate both enabling and constraining aspects where available.

## Data Availability

The datasets generated and/or analysed during the current study are not publicly available due to the potential identifiability of participants, but are available from the corresponding author on reasonable request.

## Supplementary Materials

The following supporting information can be downloaded at: https://www.mdpi.com/article/10.3390/healthcare14121803/s1. Semi-Structured Interview Guide.
