# Supplementary material for: Barriers and Facilitators of Exercise Participation Among Community-Dwelling Older Adults with Chronic Conditions: A Qualitative Study Using the COM-B Model and Theoretical Domains Framework
Source: Healthcare (Basel). 2026 Jun 22;14(12):1803. doi: 10.3390/healthcare14121803 (PMC13300240; doi:10.3390/healthcare14121803)
Supplement: Supplementary file 1 [file healthcare-14-01803-s001.zip › healthcare-4298934-supplementary.pdf]

Supplementary Material: Semi-Structured Interview Guide

Opening script

Hello, thank you for taking the time to speak with us today. My name is [name], and I am from [institution/research team]. We are conducting a study on barriers to and facilitators of physical activity and exercise participation among community-dwelling older adults with chronic conditions. The purpose of this interview is to better understand your experiences so that community health services and future interventions can be improved.

Before we begin, I would like to let you know that your participation is entirely voluntary. You may pause or stop the interview at any time. With your permission, the interview will be audio-recorded. All information will be anonymised and kept confidential, and the data will be used for research purposes only.

C0. Informed consent (brief version)

I understand the purpose, procedures, potential risks, and possible benefits of this study. I understand that participation is voluntary and that I may withdraw at any time. I understand that all research data will be anonymised and used only for scientific research.

□ Agree to participate □ Do not agree to participate (if not, end the interview)

C1. Screening and basic information

1. Age: ____ years Sex: □ Male □ Female

2. Current household registration: □ Urban □ County town □ Rural Living arrangement: □ Living alone □ Living with spouse □ Living with children/grandchildren □ Other: ______

3. Educational attainment: □ Primary school or below □ Junior high school □ High school/technical secondary school □ College or above

4. Current chronic conditions diagnosed by a medical institution or doctor (multiple responses allowed): □ Hypertension □ Diabetes □ Coronary heart disease/heart failure □ COPD/asthma □ Osteoarthritis/low back pain/leg pain □ Post-stroke sequelae □ Cancer □ Other: ______ Number of chronic conditions: ____

5. How long have you been living with your chronic condition(s)?

6. What medicines do you take regularly on a long-term basis? (It is not necessary to name the exact medications. Categories such as “blood pressure medicine”, “blood sugar medicine”, “painkillers”, or “sleep medicine” are also acceptable.) Do these medicines affect your activity or willingness to go out?

7. Do you measure your blood pressure or blood glucose before or after activity? Under what circumstances would you decide to “be less active today” or “not exercise today”?

1. Symptoms, risk management, and general activity tendency

Q1. Symptom fluctuation and risk management

When your chronic condition becomes worse or unstable, such as when you have pain, shortness of breath, fatigue, or fluctuations in blood glucose, how do you usually adjust your exercise or physical activity? Do you have your own rules or judgement criteria for deciding when it is no longer safe to continue?

Probes

What kinds of risks worry you most during exercise, for example falling, hypoglycaemia, dizziness, or something else?

Does this worry come mainly from your own experience, from a doctor’s advice, or from stories told by others?

Do you measure your blood pressure or blood glucose before or after exercise?

Under what circumstances would you decide to “do less today” or “not exercise today”?

Q2. General orientation towards movement and sitting

From your own perspective, would you say that you are someone who is “quite active and likes to move”, or someone who “prefers sitting or lying down and resting”? Why do you feel that way?

Probes

If the participant reports prolonged sitting: During those sitting periods, what are you usually doing? For example, watching television, using your phone, daydreaming, or playing cards.

2. Current life situation and daily movement patterns

Q3. Daily routines and situational influences

You have just described a typical day. Now I would like to ask a little more about several specific situations.

Probes

Do you think your exercise or activity is affected by the season or weather? For example, if it is too hot in summer, too cold in winter, or raining, how does that affect your willingness or ability to go out or be active outdoors?

Do you feel safe when going out to walk or exercise? For example, in terms of traffic, road conditions, lighting, or neighbourhood safety. What details affect you most?

During the day, when are you most likely to remain sitting or lying down for a long time? Why does that happen? For example, because of weather, habit, television programmes, or family routines?

Q4. Action planning and habit formation

Do you have any regular time points, such as after meals, after getting up in the morning, or after watching television, that usually prompt you to get up and move? If not, what do you think is missing?

What methods, if any, have you used to make yourself "have to exercise", such as checking in, exercising with someone else, rewards, or family supervision?

3. Knowledge, beliefs, and attitudes towards exercise and sedentary behaviour

Q5. Perceived benefits

What benefits do you think exercise or physical activity can bring to your health? Which benefits matter most to you? For example, controlling blood pressure or blood glucose, improving stamina, feeling less tired, sleeping better, improving mood, or having more social opportunities.

Probes

If you had to rank these benefits, which ones matter most to you?

Q6. Perceived risks and concerns

What concerns or worries do you have about the possible risks of exercise? For example, do you worry that exercise may cause excessive fatigue, trigger discomfort, or even lead to injury?

Probes

Some people say, "When you get older, you should move less and avoid tiring yourself out," whereas others say, "The less you move, the faster your body function declines." Which view do you agree with more, and why?

Q7. Perceptions of sedentary behaviour

What do you think happens to your body when you spend a long time sitting or lying down and not moving much? Have you noticed any changes in yourself?

Probes

Has anyone ever reminded you not to sit too long and to get up and move around more? Did that make much difference?

Q8. Confidence in correct exercise methods

Do you feel that you know the "correct" way to exercise, such as warming up, controlling intensity, breathing properly, or using the right movements? Which movements are you most worried about doing incorrectly? Who would you like to teach you?

Q9. What counts as "real exercise"?

Do you think housework, caring for grandchildren, or walking to buy groceries counts as exercise? What is your own standard for "effective exercise"?

Probes

Have you ever heard people say things like, "At your age, you should not overdo it"? How has that affected you?

4. Facilitators and barriers based on concrete experience

Q10. Positive experiences and maintenance

Have you ever had a period when you consistently took part in a new form of exercise or physical activity? If so, what helped you keep going? Did you have any especially positive experiences?

Q11. Interruption or non-participation

If you have ever tried exercise but later stopped, what were the main reasons you could not continue? If you have never taken part in any exercise programme, what do you think are the main reasons?

Q12. Unpleasant experiences during activity

During exercise or activity, have you experienced discomfort such as pain, breathlessness, or marked fatigue that made you want to stop? Do such feelings make you less willing, or less confident, to exercise the next time?

Q13. Specific advice from professionals

Have doctors or community health service staff ever given you specific exercise advice, such as recommended intensity, frequency, contraindications, or monitoring methods? Do you think such advice was practical and feasible?

Probes

Have you ever received conflicting advice from different doctors, such as "move more" versus "rest more"? How did you decide whom to follow?

If you have attended chronic disease management, rehabilitation training, health education classes, exercise guidance, or follow-up services provided by a hospital or community, which parts were most useful and which were not suitable for you?

If you have not attended any of these services, what were the main barriers, such as not knowing about them, distance, cost, lack of time, fear of risks, or having no one to guide you?

Q14. Restarting after interruption

How do you usually arrange your exercise? If you stop for a period of time, how do you usually start again?

5. Physical and psychological capability, and disease-related limitations

Q15. Current physical condition and activity limitations

Given your current physical condition, which symptoms or functional problems most affect your daily activity or exercise, such as walking, climbing stairs, doing exercises, or going out? To what extent do they affect you?

Probes

When you sit for too long, do you become more uncomfortable and therefore want to get up and move, or does movement make you feel even more uncomfortable so that you would rather remain seated? Which is closer to your experience?

Q16. Confidence and capability

Do you feel that you have the ability to start and maintain an exercise routine? How confident are you that you can exercise safely and correctly?

Probes

Do you worry about falling, twisting an ankle, or triggering something like a heart problem during exercise? To what extent does that worry affect your willingness to move?

Q17. Judging whether to continue or stop

When your blood pressure, blood glucose, or pain fluctuates, or when you feel unwell, how do you decide whether to continue or stop exercising? Have you ever experienced an adverse event related to exercise?

If you were hospitalised, had an acute exacerbation, or had a relapse in the past year, how did your activity level change after discharge? What helped you return to your previous level, or what prevented you from doing so?

Q18. Coping with warning symptoms

If you experience chest tightness, marked breathlessness, dizziness, palpitations, leg weakness, cold sweats or symptoms suggestive of hypoglycaemia, or a sudden worsening of pain while exercising or walking, what would you usually do? Would you carry medicine or sugar with you, or contact family members? Have such experiences made you more afraid to exercise in the future?

6. Social influence from family, peers, and healthcare professionals

Q19. Family attitudes and support

What attitudes do your family members, such as your spouse or children, have towards your daily activity and exercise? Do they support or encourage you to exercise? What practical help, if any, do they provide?

Probes

Do you currently have responsibilities for caring for your spouse, grandchildren, or other family members? Do these responsibilities take up your exercise time or leave you too tired to exercise?

Q20. Peers and social norms

Do you have friends or peers who exercise with you? Is exercising with other people important to you? What do people around you, such as neighbours, friends, or others of a similar age, generally think about exercise for older adults?

Q21. Advice from healthcare professionals

Have doctors, nurses, or community healthcare staff ever advised you to do appropriate exercise? For example, have they reminded you to get up and walk regularly, or prescribed exercise? How willing have you been to follow such advice?

Probes

Have doctors or nurses ever given you specific and actionable recommendations, such as exercise intensity, frequency, precautions, or risk monitoring? Would you follow such advice? Why or why not?

Q22. Doctor–patient interaction and trusted information sources

How much influence do healthcare professionals’ recommendations have on you? Which information sources do you trust most, for example hospitals, community services, short videos, or family and friends? What kind of advice is most likely to make you take action?

7. Physical environment, resources, and broader support conditions

Q23. Neighbourhood environment

Do you think your residential community and nearby environment are convenient for exercise? For example, are the roads flat and easy to walk on? Are there stairs or ramps?

Q24. Facilities and exercise spaces

Are there parks, outdoor fitness equipment, or sports centres nearby for you to use? Do these environmental conditions affect your willingness to go out and exercise?

Q25. Mobility aids and environmental safety

Do you use a cane, walker, knee support, or ankle support when going out to exercise? Which environmental details affect your sense of safety most, for example steps, ramps, road surfaces, lighting, benches, toilets, or the distance needed to cross the road?

Probes

Given your current physical condition, which places feel suitable or unsuitable for you? Where do you feel most unsafe?

Q26. Activity atmosphere in institutions

If you often attend a day-care centre, senior university, or community exercise station, do you feel that the atmosphere there encourages movement, or is it more focused on sitting and chatting?

Q27. Costs and practical burdens

What kinds of costs or practical difficulties are associated with exercise for you? For example, financial costs, travel distance, transportation, or time constraints. Are any of these concerns particularly important to you?

Probes

If exercise classes or rehabilitation programmes were partly reimbursed by health insurance, or provided free of charge or at low cost by the community, would you be more willing to take part? At present, what are the main financial or time-related barriers?

Q28. Relative priority of exercise

Compared with medical expenses and family caregiving responsibilities, where does exercise rank in your life in terms of priority? Why?

8. Preferences for ideal exercise and sedentary behaviour reduction interventions

Q29. Preferred forms of activity

If you could choose freely, what would your ideal form of exercise be? Would you prefer to exercise alone or with others? Would you rather exercise outdoors, such as in a park, or indoors, such as in an activity centre? Do you have preferences regarding frequency and duration?

Probes

If the goal were simply to "move a little more and sit a little less" in daily life, such as standing up during television breaks, walking while talking on the phone, or doing seated leg lifts, which of these would feel realistic for you?

Q30. Support needs and preferred intervention format

What kinds of support would you need in order to be more willing to start and continue exercising, for example professional guidance, companionship, family encouragement, access to equipment or venues, or financial support?

Probes

If doctors, community staff, or exercise instructors worked with you to co-design a personalised plan and also helped you reduce sitting time, what kind of involvement would you find most acceptable, for example small-group discussion, questionnaire-based feedback, or trial participation followed by regular feedback?

Q31. Views on traditional forms of exercise

What do you think about traditional forms of exercise such as Tai Chi, Baduanjin, or square dancing? Do you feel that these are more compatible with your identity and way of life?

Q32. Use of digital tools

Do you use a pedometer, mobile phone, smart bracelet, or mini-programme to monitor activity? Do you find these convenient? Which functions are most useful to you, and which do you dislike most?

9. Overall reflection and recommendations

Q33. Main barriers and key motivators

For you personally, what is the single biggest barrier to exercise at present? What is the strongest factor that motivates, or would motivate, you to take part in exercise?

Q34. Recommendations for society and institutions

What suggestions would you make at the social or institutional level? For example, what support or measures would you like communities, healthcare institutions, or government departments to provide in order to help older adults with chronic conditions participate in exercise more effectively?

Q35. Additional comments

We have discussed many topics today. Is there anything else you would like to add? Is there anything important related to this topic that we did not ask about but that you think should be discussed?

Q36. Structural and resource-related influences

Apart from personal reasons, what kinds of broader systems or resources do you think influence exercise participation among older adults, for example venues, costs, health insurance, community programmes, or transport? If you could change one thing most urgently, what would it be?
